# CcpNmr AnalysisScreen, a new software programme with dedicated automated analysis tools for fragment-based drug discovery by NMR

**DOI:** 10.1007/s10858-020-00321-1

**Published:** 2020-07-07

**Authors:** Luca G. Mureddu, Timothy J. Ragan, Edward J. Brooksbank, Geerten W. Vuister

**Affiliations:** Present Address: Department of Molecular and Cell Biology, Leicester Institute of Structural and Chemical Biology, University of Leicester, Henry Wellcome Building, Lancaster Road, Leicester, LE1 7HN UK

**Keywords:** Screening, Fragments based drug discovery, NMR, FBDD, CCPN, CcpNmr software

## Abstract

Fragment-based drug discovery or FBDD is one of the main methods used by industry and academia for identifying drug-like candidates in early stages of drug discovery. NMR has a significant impact at any stage of the drug discovery process, from primary identification of small molecules to the elucidation of binding modes for guiding optimisations. The essence of NMR as an analytical tool, however, requires the processing and analysis of relatively large amounts of single data items, e.g. spectra, which can be daunting when managed manually. One bottleneck in FBDD by NMR is a lack of adequate and well-integrated resources for NMR data analysis that are freely available to the community. Thus, scientists typically resort to manually inspecting large datasets and relying predominantly on subjective interpretations. In this manuscript, we present CcpNmr AnalysisScreen, a software package that provides computational tools for automated analysis of FBDD data by NMR. We outline how the quality of collected spectra can be evaluated quickly, and how robust workflows can be optimised for reliable and rapid hit identification. With an intuitive graphical user interface and powerful algorithms, AnalysisScreen enables easy analysis of the large datasets needed in the early process of drug discovery by NMR.

**Electronic supplementary material:**

The online version of this article (10.1007/s10858-020-00321-1) contains supplementary material, which is available to authorized users.

## Introduction

**Fig. 1:**
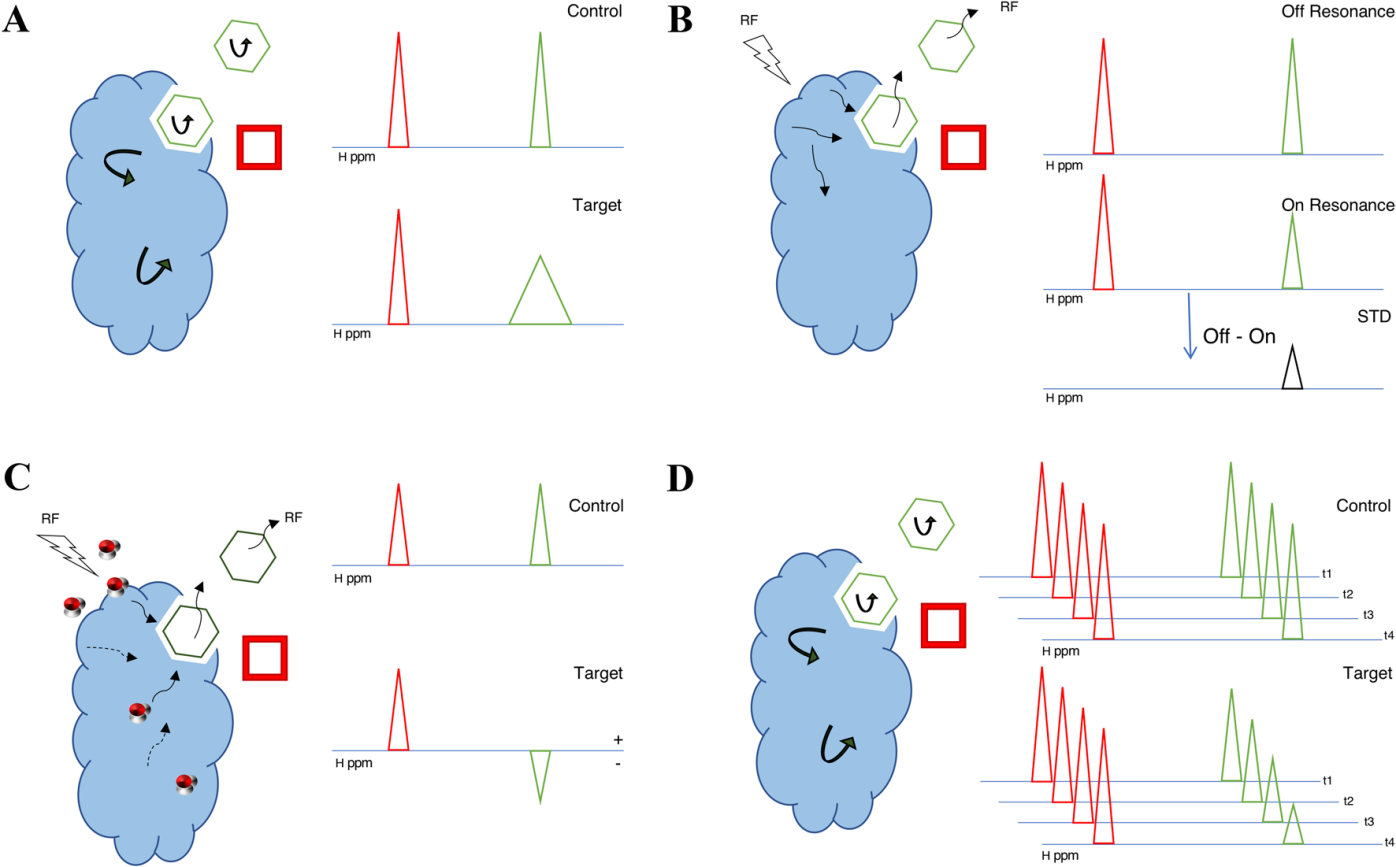
Ligand-detected NMR methods. Common techniques for detecting ligand binding (Sugiki et al. 2018) to a large macromolecular target (blue motif). The binding and non-binding compounds (small molecules) are displayed as a green hexagons and red squares, respectively **a** ^1^H Relaxation-edited experiment. The peaks of both compounds in the control spectrum are characterised by narrow resonance lines. In the presence of a target, a binding compound partially acquires the NMR properties of the macromolecule, resulting in a broadening of its resonance line (green peak). The effect does not affect a non-binding compound. **b** In the on-resonance experiment of a saturated transfer difference (STD) experiment, a saturating RF field is applied to the target and saturation is transferred to the binding compound, resulting in a slightly lower intensity of its resonance line. In the off-resonance control experiment no such effect occurs; consequently, only the resonance of the binding compound will be visible in the STD spectrum. **c** In the WaterLOGSY experiment saturation is transferred to the target through saturation of the bulk water molecules and passed on to the binding compound. Its resonance line in the spectrum in the presence of the target will have the opposite sign compared to the control spectrum. **d** In the T_1_ρ experiments a series of spectra are recorded with different relaxation durations. For the binding compound, spectral intensities will attenuate at a faster rate compared to the non-binding compound

Over the years, the versatility of NMR as a non-destructive and adaptable analytical tool has encouraged the development of multiple fragment-based drug discovery (FBDD) approaches by NMR (Dias and Ciulli (2014). Nowadays, it is possible, albeit not frequently done, to conduct the entire drug discovery process by NMR: from hit detection and binding site identification to the determination of the ligand orientation and hit optimisation. A meticulous examination of recent FDA-approved drugs and drugs in clinical stage studies, indicates a substantial contribution of various NMR-based techniques to the whole drug discovery process (Petros et al. 2006; Szlávik et al. 2019; Schoepfer et al. 2018; Erlanson et al. 2016a). Assuming the target of interest has already been identified, hit identification is usually the first step in the drug discovery process and this is the aspect we concentrate on in this article. This can be achieved by NMR using a number of common ligand-detected NMR methods (Dias and Ciulli 2014), namely ^1^H-relaxation-edited (commonly called ^1^H), saturation transfer difference (STD) (Mayer and Meyer 1999), WaterLOGSY (Dalvit et al. 2000) (Fig. 1a), and alternative relaxation experiments (T_1ρ_, T_2_). In addition, a number of complementary techniques, i.e. target immobilised NMR screening (TINS) Vanwetswinkel et al. (2005), spin label analysis (Jahnke 2002), paramagnetic relaxation enhancement (PRE) (Guan et al. 2013) and ^19^F experiments (Dalvit and Vulpetti 2012) have been successfully used in the primary hit identification process.

All direct ligand-observed NMR methods rely on the differential molecular properties of the target and ligand, strategically recording only ligand signals while suppressing the detection of target signals thus allowing for a significant reduction of spectral crowding.

A small-molecule ligand engaged in a fast-exchange complex with a macromolecule partially acquires the spectroscopic NMR properties, e.g. T_1_/T_2_ relaxation and ^1^H-^1^H cross-relaxation rates, of the macromolecule. When there is a sufficiently large molar excess of the small molecule ligand, this typically results in the detection of chemical shifts of the ligand free-state, but with modified relaxation properties more reminiscent of the bound state (Campos-Olivas 2011) (Fig. 1a). For example, small molecules tumble fast in solution and hence their NMR resonance lines are characterised by long transversal relaxation times (T_2_) that result in narrow lines. In contrast, when bound to a slowly tumbling macromolecules the NMR lines of the small molecule are significantly broader. Therefore, in the case of fast exchange of the small molecule between the free and bound states, its NMR signals will become broadened (Fig. 1a).

The saturation transfer difference (STD) experiment relies on the efficient spin-diffusion of saturated proton magnetisation in the macromolecule through measurement of the so-called “on-resonance” and “off-resonance” experiments. In the “on-resonance” experiment, selected ^1^H resonances of the macromolecule that are non-overlapping with those of the ligand are saturated using a train of RF pulses. The saturation propagates rapidly through the macromolecule and to the bound ligand as a result of efficient intramolecular and intermolecular ^1^H-^1^H cross-relaxation, respectively (Lepre et al. 2004) (Fig. 1b). As the ligands are in rapid exchange between their bound and free states, they maintain their saturated state resulting in attenuated or even absent signals in the resulting “on-resonance” spectra. In the “off-resonance” control experiment, the macromolecular resonances are not saturated resulting in signals with original intensities. Subtraction of the “off-resonance” spectrum from the “on-resonance” spectrum yields the STD spectrum, in which only saturated ligand resonances will be observable (Fig. 1b). The signals of the macromolecule will be minimal or absent, as a result of the much smaller concentration of the latter in comparison to the ligand, thus greatly simplifying spectral analysis.

In an alternative approach, the so-called WaterLOGSY experiments (Dalvit et al. 2000, 2001) (Fig. 1c), the ligand and macromolecular target are saturated indirectly through the bulk water magnetisation. The saturation is transferred from the bulk water to the ligand through several mechanisms, in particular by direct ^1^H-^1^H intermolecular cross-relaxation between water molecules in close proximity to the binding pocket and the bound ligand. Alternative mechanisms include the direct exchange with macromolecular NH and OH protons within the binding site and the ligand, or indirectly, through a spin-diffusion mechanism. In both cases, NMR properties of the bulk water are transferred to the bound ligand, and the resulting spectrum displays inverted signals for bound ligands compared to the unbound ligands (Fig. 1c). The detection of ligands that bind to macro-molecules with a relatively low density of protons might benefit from the WaterLOGSY technique (Jahnke 2002). Furthermore, WaterLOGSY experiments have displayed higher sensitivity for detecting binding molecules compared to STD experiments when used to screen very large biomolecules at low concentrations (Antanasijevic et al. 2014). Antanasijevic et al*.* believed that this is caused by the higher concurrent (direct and indirect) saturation of various sites in the binding complex (Antanasijevic et al. 2014).

A third approach exploits the altered T_1_/T_2_ relaxation properties of ligands that bind to a macromolecular target (vide supra). In the so-called ^1^H-relaxation-edited experiment, also referred to as the T_1ρ_ experiment, a series of spectra are recorded in which the ligand signals are subjected to varying durations (typically in a range of 1 to 200 ms) of transverse relaxation, i.e. either as R_2_ or R_1ρ_. Bound ligands will exhibit faster R_2_ or R_1ρ_ rates, i.e. shorter T_2_ or T_1ρ_ relaxation times, and their signals will be significantly attenuated in the spectra compared to ligands that do not bind to the macromolecular target (Fig. 1d).

In spite of all the powerful NMR experiments used for NMR-based FBDD (Sugiki et al. 2018), inefficient evaluation of the primary hit screening data can disrupt or postpone any of the later phases, such as binding site identification and hit optimisation (Fig. S1).

Primary screening is routinely performed manually by comparing spectral information derived from thousands of STD, WaterLOGSY and relaxation-edited experiments. Manual analysis of these data inevitable results in human errors or subjective inconsistencies, in addition to problems arising from commonly occurring experimental errors, such as improper alignment and scaling of spectra. The latter are detrimental to the accurate assessment of any datasets, whether manual or automated. Even when using computational routines, several inherent difficulties to the data analysis process still remain. The different nature of each NMR screening experiment translates into fundamentally different spectral patterns. Consequently, it requires robust algorithms, such as those employed for peak detection or peak matching, that ideally require no fine tuning of algorithms via adjustable parameters as this would slow-down, complicate and reduce the reproducibility of whole data analysis. Accurate peak detection is also fundamental for the generation of the most optimal mixtures on the basis of the library of spectra of the compounds, as subsequent deconvolution of their spectra is a key step in the identification of potentially binding compounds.

Currently, only a limited number of tools that provide support for NMR screening exist, such as Bruker TopSpin (TopSpin) or MestreLab MNova Screen (Peng et al. 2016), both of which are often not affordable for occasional or academic users. Alternatively, NmrGlue (Helmus and Jaroniec 2013), a freely available collection of NMR library functions, could serve as the building blocks for creating stand-alone custom scrips for expert users, but to the best of our knowledge no such efforts have been documented. In this manuscript we introduce the CcpNmr AnalysisScreen software programme, or AnalysisScreen for short, which is part of the Analysis version-3 software suite (Skinner et al. 2016) as an alternative data analysis and inspection platform. AnalysisScreen aims to facilitate the hit identification process by offering a set of tools for streamlined inspection of spectral data, automation of common processing and analysis workflows. As a result, AnalysisScreen assists in both qualitative and quantitative inspection of NMR data, reducing false negatives (wrongly missed or rejected hits) and false positives (wrongly accepted hits). The AnalysisScreen core is implemented with the requirements of speed and customisation in mind, thus offering users a platform capable of easy adaptations, following any future NMR methods that might emerge.

## Materials and methods

### Computational libraries

AnalysisScreen is written in the Python 3.6 programming language. Synthetic datasets, implemented algorithms, routines and macros, were written using the open-source scientific libraries such as Numpy, ScyPy (Taschini 2008), Sci-kit Learn (Pedregosa et al. 2011) and Numba which are included in the main CcpNmr environment (Skinner et al. 2016). Numba (Lam et al. 2015) has been used to improve the speed of repeated and time-consuming routines, such as peak picking. Pandas (McKinney 2011), has been used mainly for importing, parsing, exporting and filtering metadata. PyQt5, PyQtGraph (Campagnola), Matplotlib (Hunter 2007) and Seaborn (Waskom et al. 2017), have been employed for plotting and results analysis as well as for building custom widgets into the main programme.

The core code and concept of the NmrMix simulated-annealing algorithm (Stark et al. 2016), including its scoring function, were used to implement the mixture analysis module included in CcpNmr AnalysisScreen. Although the crucial simulated-annealing algorithm steps were unaltered as in the original package, it has been speed-optimised. We also included the ability to preserve the best-scored mixtures and included an option for their use as input for subsequent generations, while retrieving them if ameliorated solutions could not be achieved.

The peak picker algorithm used for analysing these datasets was based on the method described by Billauer (2012). The Algorithm has been optimised to handle larger NMR datasets using Numba’s properties, and inserted extra filters, such as masked regions (to be ignored from the analysis) and removal of local minima. The positive noise threshold is used as the delta value in the peak picker.

Positive and negative noise thresholds are estimated automatically as follows:

1 $${\text{N}}_{{{\text{Th}}}} = \alpha \sigma {\text{N}}* {\text{N}}_{{{\text{Max}}}}$$

where N is a defined downfield region of the spectrum, by default 10% of the total datapoint count; σ is its standard-deviation and α is the adjustment factor. N_Min_, is used instead of N_Max_ to calculate the negative threshold.

Negative and positive noise threshold values were used to calculate the Signal-to-Noise ratio as

2 $${\text{SN}}_{{{\text{Ratio}}}} = {\upalpha }*\frac{{\text{S}}}{{{\text{N}}_{{{\text{Max}}}} - {\text{N}}_{{{\text{Min}}}} }}$$

where S is the peak height and α is the adjustment factor. N_Max_ and N_Min_ are the positive and negative noise threshold values.

### Scorings

Matching and relative scores for hit identification were calculated as

3 $${\text{S}}_{{{\text{Rel}}}} = \left| {{\text{A}}_{{{\text{Med}}}} } \right|{\text{*A}}_{{{\text{Tot}}}}$$

where A_Med_ represents the median for the absolute observations (peak heights or Δppm positions for matching scores) and A_Tot_ the total count. If only two values are present in the array, then only the minimum value is taken:

4 $${\text{S}}_{{\text{Rel*}}} = { }|{\text{A}}_{{{\text{Min}}}} |{\text{*A}}_{{{\text{Tot}}}}$$

Hit scores were normalised to values in a range 0–100 by:

5 $${\text{S}}_{{{\text{Tot}}}} = {100}*\frac{{{\text{S}} - {\text{S}}_{{{\text{Min}}}} }}{{{\text{S}}_{{{\text{Max}}}} - {\text{S}}_{{{\text{Min}}}} }}$$

where S are the relative scores calculated using Eqs. 3 and/or 4.

### Testing datasets

To evaluate AnalysisScreen’s capabilities we used two types of spectral datasets. The first was artificially created, and it is referred to as “simulated”; whereas the second dataset consisted of a total of 2070 spectra provided by our industrial collaborators as part of an actual experimental screening trial. It is referred throughout the manuscript as “experimental”.

Simulated spectral datasets were generated using in-house written scripts (macros) in Python, employing the AnalysisScreen Python environment. Using these macros, we were able to create an arbitrary number of spectral peaks at random positions and heights, and with Lorentzian line shapes with varying linewidths. To test the dependency of correctly identifying a hit on the Signal-to-Noise (S/N) ratio, we simulated an STD spectrum for 100 compounds and recreated 300 randomly generated copies at various S/N ratios. For simplicity, only one peak per spectrum was created at a random position. The peak picker routine was expected to find a total of 100 known true positive peaks and 100 true negatives. Total true negatives were set arbitrarily to 100 to avoid an unbalanced dataset. Molecule structures, including SMILES, and other chemical properties were randomly created and assigned to the spectra. All simulated datasets and metadata generated for this work were used only for testing or demonstration purposes and have no biological significance.

The experimental dataset consisted of a library of 1760 small-molecule compounds, for which a processed one-dimensional reference spectrum was provided in Bruker format. From this library, 1548 fragments had been used to create 310 samples containing four to five, randomly selected small ligands at ~ 200 μM each and an unnamed target at ~ 4 μM. A processed STD spectrum for each sample was provided. Although all the crucial data needed for the assessment of the AnalysisScreen routines was available, the biological information and detailed experimental conditions were confidential and not shared with us.

## Results and discussion

### Parsing and importing NMR data and metadata

**Fig. 2:**
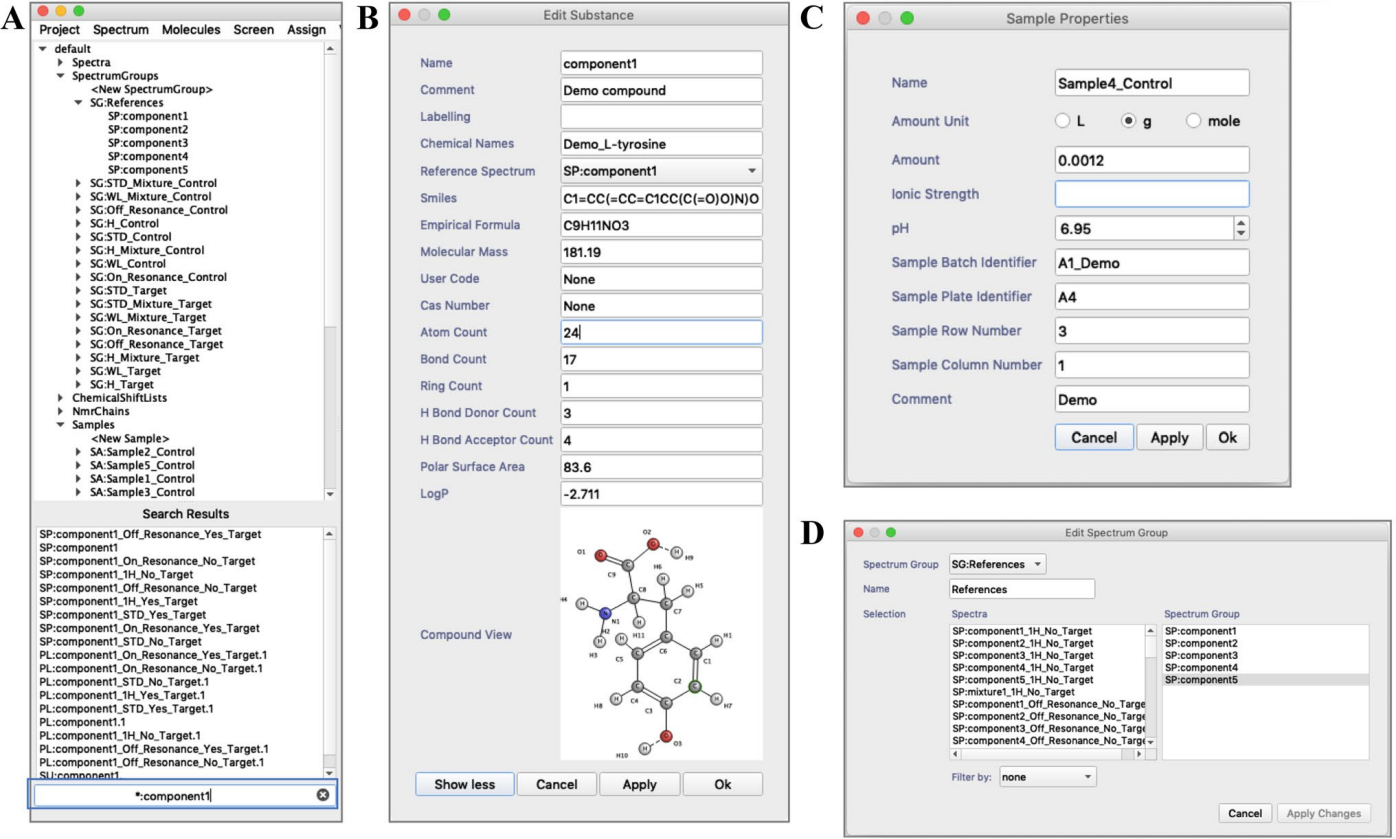
CcpNmr AnalysisScreen sidebar and various pop-ups. **a** Screenshot of the sidebar state after parsing and loading an Excel file containing spectral metadata. Objects are automatically created and are listed on various branches. The regex-enabled search widget (blue rectangle) allows for quick scanning of project metadata through the tree, an essential feature when handling several hundred entries of a typical NMR screening dataset. **b** Small molecule metadata are stored into the CcpNmr software as Substances. Substances are a representation of chemical properties of the reference compound. They can be visualised and edited in the Substances pop-up. If SMILES are provided, molecular structures are also shown in this window. **c** The Samples properties pop-up enables users to insert and edit information regarding particular experimental conditions, such as concentration and pH or other sample identifiers. **d** The SpectrumGroup editor pop-up allows users to quickly and easily group spectra using drag-and-drop features. SpectrumGroups can be displayed as single entities in displays or be used as input data for several tools throughout the programme

Typically, an NMR based FBDD screening experiment requires the handling of a large volume of spectral data and metadata. To address this problem, we included in AnalysisScreen the option to use spreadsheets in Excel format as a data loading mechanism. The programme can natively read, parse and load files with multiple sheets (Fig. S2A–B), where column-based keywords define the relevant pieces of information. Upon parsing and importing into AnalysisScreen, commonly used parameters and information associated within a sample, e.g. different experimental conditions, are immediately available within the sidebar of the AnalysisScreen programme (Fig. 2a). All metadata is retained with the relevant CcpNmr object, such as experiment types of spectra or SMILES and other chemical properties of molecules, named Substances in the programme nomenclature. All objects used for screening analysis can also be graphically inspected, edited or deleted using dedicated pop-ups (Fig. 2b–d).

To further simplify the data analysis preparation, the data loader also includes an automatic path recognition ability so that specifying the absolute spectral data locations is no longer required. In addition, spectra can be automatically grouped into so-called SpectrumGroups; these are user-defined collections of spectra, designed in such a way that multiple routines can be applied uniformly to all their items. SpectrumGroups follow the same philosophy of single spectra when it comes to visualisation, and can, therefore, be displayed and manipulated as single entities. Samples, SampleComponents, Substances, SpectrumGroups and SpectrumHits objects are internally connected, forming the underpinning core objects of the AnalysisScreen programme (Fig. S2C). AnalysisScreen maintains the same organisational working areas of CcpNmr AnalysisAssign (Skinner et al. 2016), called modules. Modules are containers designed to visualise, inspect and perform actions on all types of data the project might contain.

### Assessment of spectral quality by PCA decomposition

**Fig. 3:**
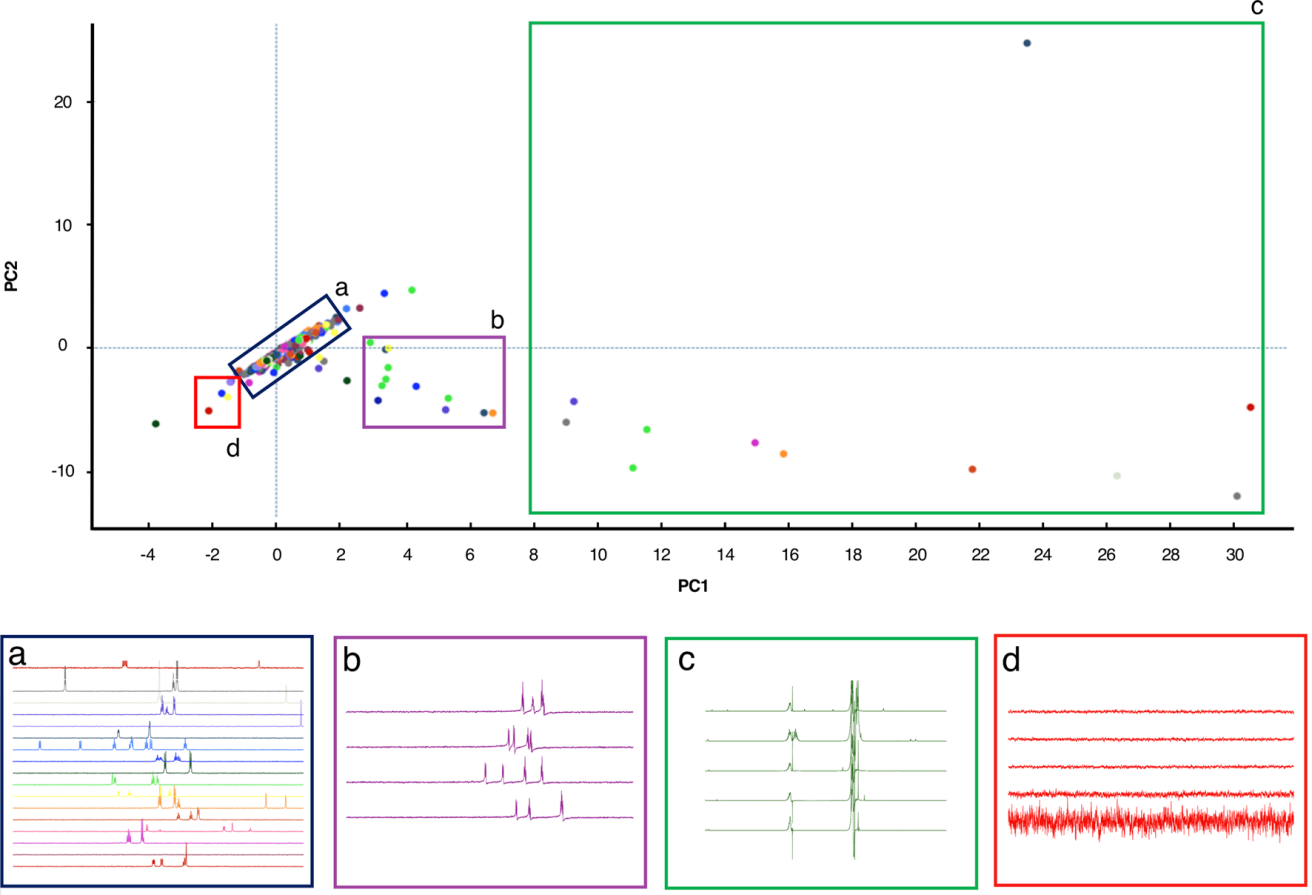
Principal component analysis (PCA) of 1760 reference spectra. Most of the spectra were uniformly grouped around the PCA origins, (blue rectangle, panel **a**); for spectra in the region 3 < PC1 < 7 (purple rectangle, panel **b**) large phasing errors were observed; the spectra in the region PC1 > 8 (green rectangle, panel **c**) appeared highly distorted, probably due to inadequate solvent suppression. Finally, spectra presenting only noise were discovered in the region indicated by the red square (panel **d**)

Commonly, NMR primary screening studies rely on a collection of one-dimensional spectra acquired for each compound in the screening library, called the reference spectra or reference library. The reference library is typically recorded in an automated fashion and its data are used throughout the analysis. Therefore, ensuring its suitability by filtering out any potentially compromised spectra is essential. Nonetheless, inspecting spectra individually for large libraries can be a time-consuming task. Principal Component Analysis, PCA (Stoyanova and Brown 2001), can be used for the assessment of spectra, without pre-knowledge of spectral line shapes or other peculiarities. AnalysisScreen offers an integrated PCA decomposition module, capable of effortlessly performing a PCA on large libraries. Figure 3 displays the result of a PCA analysis performed on a SpectrumGroup consisting of 1760 experimental reference spectra. The result of this analysis shows a high variance dispersion among the first two PCA components, enabling quick identification of any outliers. Intriguingly, we could identify several groups of spectra that displayed similar processing defects or other spectral imperfections (Fig. 3, sections b, c and d), such as phasing artefacts, inadequate solvent suppression or even the absence of signal data all together. Also, very high values of the Q-Score, a metric commonly used for evaluating variations outside of the PCA model (Mujica et al. 2011), easily identified most of the irregular spectra (Fig. S3A).

In the AnalysisScreen PCA module, each data-point in the PCA space is linked to its corresponding spectrum, so it can be easily accessed, inspected, removed from the project, or corrected using other tools such as pipes (vide infra) present in the programme. Furthermore, the decomposition module allows principal component vectors to be displayed and offers the possibility to create new simulated spectra or export the various scores (Fig. S3B).

### Mixture optimisations

Following the quality assessment of the reference library, its reference spectra form the basis for generating mixtures based on their peaks. In fact, for reducing the experimental resources required for NMR-based screening, i.e. samples, NMR time, etc., a common approach is to analyse several compounds simultaneously against a target in a so-called mixture, which should be carefully designed to minimise spectral overlap. Manually generating random mixtures can result in overcrowded spectra, which are difficult to interpret, error prone and time-consuming when it comes to deconvoluting single signal entities to identify possible binders. AnalysisScreen includes optimisation tools that allow the user to create and edit mixtures, thus minimising spectral overlaps. The core engine of the AnalysisScreen mixtures module uses the powerful NmrMix simulated annealing algorithm (Stark et al. 2016). However, we significantly boosted the execution speed of key numerical routines by converting on “the-fly” the original Python code in a compiled machine language. The mixture generation tool also guarantees that mixtures and scores are internally preserved during all iterations and eventually the best-scoring solutions are presented to the users. AnalysisScreen can create mixtures *de-novo* starting from reference spectra, but it can also be used to score existing mixtures, such as the one provided by our collaborators. The latter was generated randomly without any further optimisation.

We assessed the mixture generation tool with an initial 1000-iterations calculation and calculated the total overlap score for each iteration (Fig. S4A). The evolution of the simulation shows the pattern of this stochastic algorithm, with the overlap score reaching several minima just above a value of 1250, which is notably better than value of 1381 obtained for the original randomly created mixtures. However, some iterations displayed considerably inferior values; those solutions were obviously discarded. To assess the influence of the size and the nature of the dataset, we divided our original input into either four or ten random SpectrumGroups and performed the calculations followed by joining the results in a single clustered output. This simple strategy showed a further progressive reduction in total overlaps and scores (Fig. S4B). Although this result is somewhat counterintuitive, we speculate that by introducing four or ten random groups, we have increased the overall randomness of the sampling algorithm with respect to relevant spectral regions of interest. Nonetheless, our findings demonstrated the importance of running a large number of iterations to establish an optimal mixture, rather than relying on a few single individual optimisations. Using the automated approach, significantly optimised mixtures were generated when compared to the original randomly generated one. Importantly, we find both a shift to lower values in the distribution of the scores of each mixture as well as a reduction in the number and lowering of the most poorly scoring mixtures, i.e. those with the most problematic overlap. It is to be expected that the latter represent the most challenging mixtures in the analysis of the data (vide infra).

### Pipelines

**Fig. 4:**
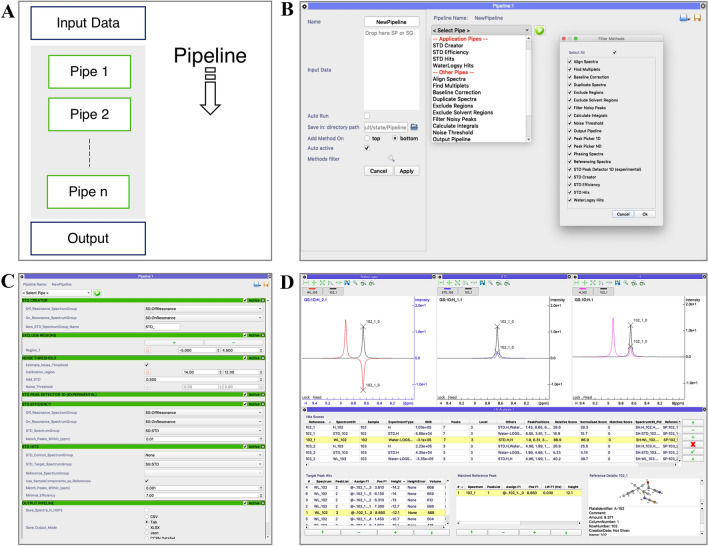
CcpNmr AnalysisScreen Pipeline and Hit Analysis module. **a** Schematic representation of a pipeline. The pipeline is able to handle SpectrumGroups as well as single spectra as the input data. Each pipe performs a dedicated action on the spectra and returns a new set of spectra which are used as input for each successive pipe. Finally, a result or report pipe provides information on performed actions. **b** Current graphical user interface for assembling and executing a Pipeline. The left side shows the available settings affecting the execution of the pipeline. Pipelines are constructed by simply selecting pipes from the main pull-down; the grey area underneath displays the selected pipes. On the right side, a pop-up is shown which can be used to customise the main selection pull-down. Pipelines can also be saved and restored, including last used parameters, as a JSON file that can be shared with other AnalysisScreen users. **c** A pipeline for STD hit identification. Each green header represents a pipe action. The pipe can be as simple as the Peak Detector, without user adjustable parameters, or a list of complex widgets such as the Noise Threshold pipe, which allows direct interaction with displayed spectra. **d** Current Hit Analysis module graphical user interface containing a report of 1000 simulated samples for three different experiment types. The Hit Analysis module allows interactive inspection and assessment of SpectrumHits showing spectra, scores and associated metadata. Furthermore, custom peak tables (bottom) allow quick navigation through the peak hits in the selected spectrum display. A summary for the sample and SpectrumHit properties is shown in the bottom right corner

The heterogeneity of NMR techniques for 1D screening, translates into the need for specific analysis workflows for each method. We addressed this by designing and implementing the AnalysisScreen pipeline module (Fig. 4a, b). It permits users to apply multiple tasks or algorithms, called pipes, to single spectra or all spectra contained in a SpectrumGroup.

AnalysisScreen features application-specific pipes, such as line broadening, WaterLOGSY and STD hit detection, as well as a set of other data manipulation pipes that are shared across all other Version-3 Analysis programmes (Skinner et al. 2016). These include but are not limited to alignment, re-referencing and phase correction. Furthermore, the pipeline architecture easily allows the addition of user-defined operations such as a bespoke pipe, (Fig. S5A–B). The pipes together form a so-called pipeline that effectively implements a user-defined workflow. Any pipeline can be saved as a JSON file for re-use or exchange with other users of the CcpNmr Analysis suite. An example of an STD analysis pipeline is shown in Fig. 4c. The pipeline consists of a set of seven simple tasks, some of which are experiment-specific, such as *STD Spectrum Creator*, *STD Efficiency*, *STD Hits*, and some of which affect generic tasks, e.g. *Noise Threshold*, *Exclude Regions*, *Peak Detector* pipes dictate the picking peaking. And finally, there is the *Output Pipe.* Each of these pipes is fully documented in the available tutorials within the software. SpectrumHits, defined as a detectable and identifiable signal that has changed relative to its control, can be accessed and inspected graphically by the Hit Analysis module (Fig. 4d). This module allows interactive navigation to spectra and peaks for the best-matched references and SpectrumHits. Furthermore, the main table allows quick and straightforward assessment of the best results by rank-order examination of several scores and display of all associated hit metadata.

**Fig. 5:**
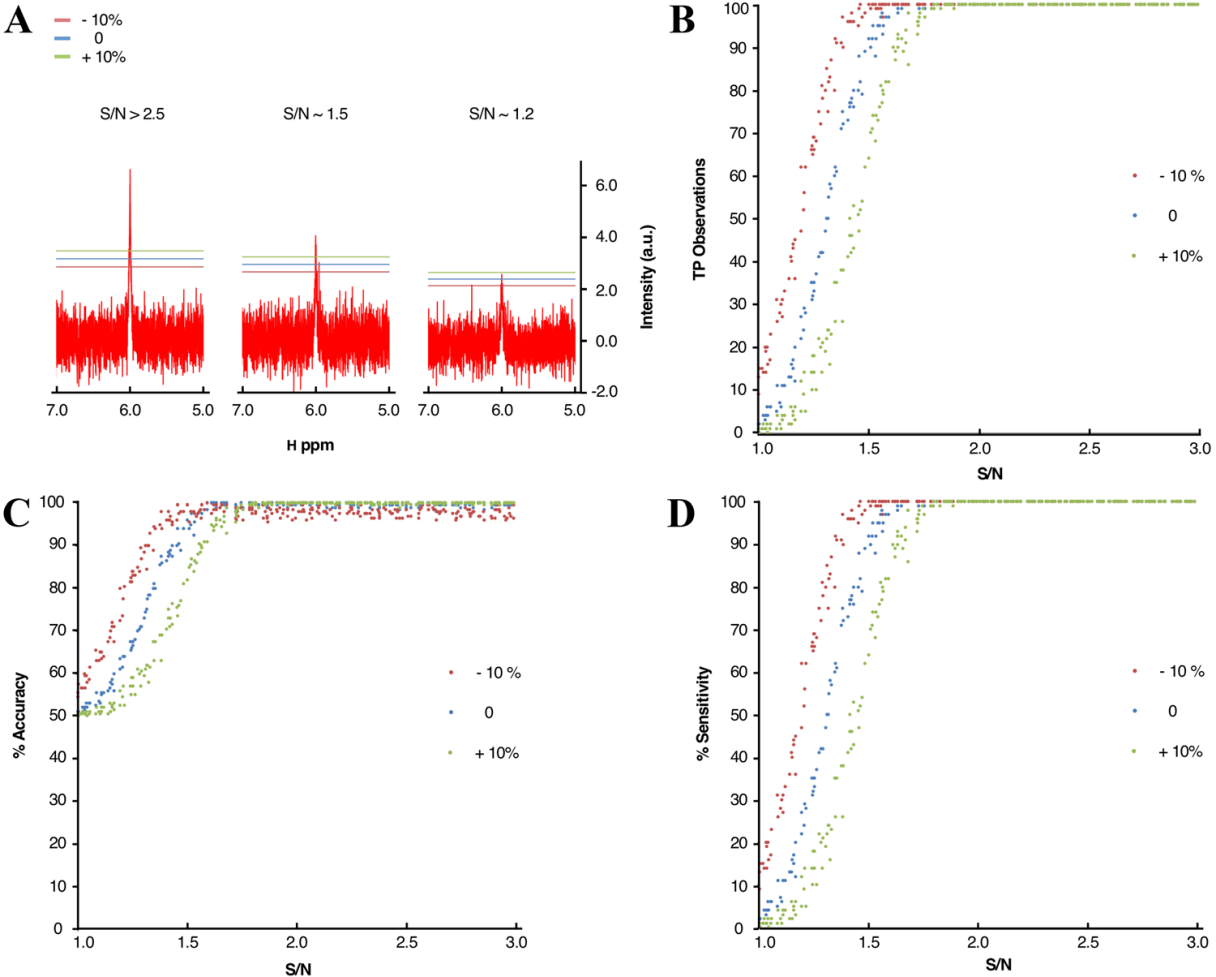
Peak and hit detection assessment using simulated spectra. **a** Simulated ^1^H spectra at different signal-to-noise ratios and estimated positive noise thresholds calculated using Eq. 1, with *α* set to 1.5 (blue), relative adjustment N_Th+10_ = + 10% N_Th_ (green) and N_Th-10_ = − 10% (red). The left panel shows typical spectral peaks with an S/N greater than 2.5. Peak intensities are well above threshold values and peaks are correctly identified. At around a S/N of 1.5, most of the peaks are still identified, although a larger number of artefacts can be mistakenly included as real peaks. At very low S/N it is generally difficult to distinguish genuine peak shapes from the spectral noisy distortions. **b** Total count of correctly identified observations for 100 simple spectra simulated at over 20,000 different S/N variations. **c** Total accuracy for the peak picker on simulated spectra at different delta values. Accuracy (A) was defined as A = (TP+TN)/(TP+FN+FP+TN). **d** Total sensitivity for the peak picker on simulated spectra. Sensitivity (S) was calculated as S = TP/(TP + FN), with TP, TN, FP and FN denoting true positive, true negative, false positive, and false negative values, respectively

Pipelines were initially tested on a series of small datasets simulating typical spectral patterns for STD, WaterLOGSY, and ^1^H-relaxation-edited experiments. For each of these experimental screening data types the SpectrumHits, were identified correctly (Fig. S6). We then created a larger dataset of simulated spectra at various Signal-to-Noise ratios (S/N) to determine the S/N regime for which observations could be accepted reliably as True Positive (TP) hits (Fig. 5a). Using these simulated spectra, we also evaluated the peak picker algorithm for its accuracy and sensitivity to correctly locate and distinguish the spectral signal from the noisy part of the spectrum. Using an in-house noise level threshold detection routine (Eq. 1), it was possible to detect over 90% of TP observations down to an estimated S/N of ~ 1.5 (Figs. 5b and S7A). Decreasing threshold parameters in an attempt to include more TP observations at lower S/N resulted in a decrease in general accuracy and precision, which is, obviously, not favourable (Figs. 5c, d and S7A–D). Analysis of the receiver operating characteristic (ROC) curve (Fig. S7D) shows the calculated threshold value to be located in the most favourable part of the ROC curve, also suggesting it can be used as a reliable threshold for the automatic peak picking routine.

**Fig. 6:**
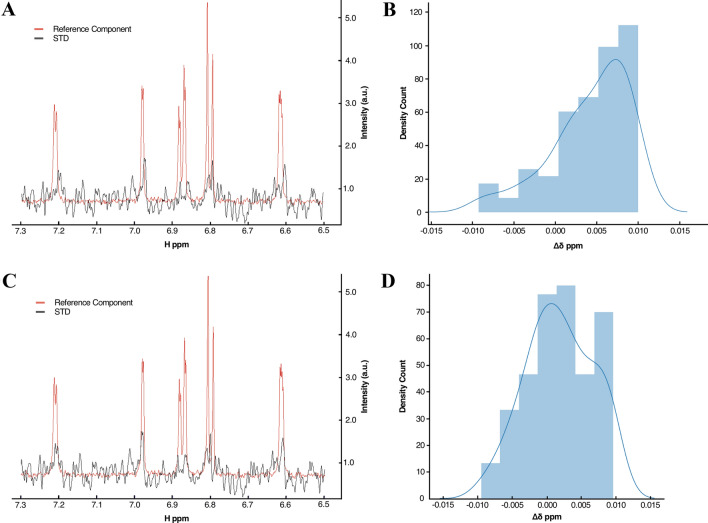
Re-referencing of spectral datasets. **a** and **c** show an example of an STD SpectrumHit and its best-matched reference before and after applying a re-referencing pipe. **b** and **d** illustrate peak shift distributions of experimental STD spectra to their reference spectra before and after a re-referencing pipe was applied. The maximum of the distribution, ~ 0.0075 ppm, (from Fig. 6b), was used to calculate the total adjustment needed to re-reference the STD spectra to their references. **d** New distribution after the adjustment was applied, with a maximum centred around ~ 0.000 ppm

**Fig. 7:**
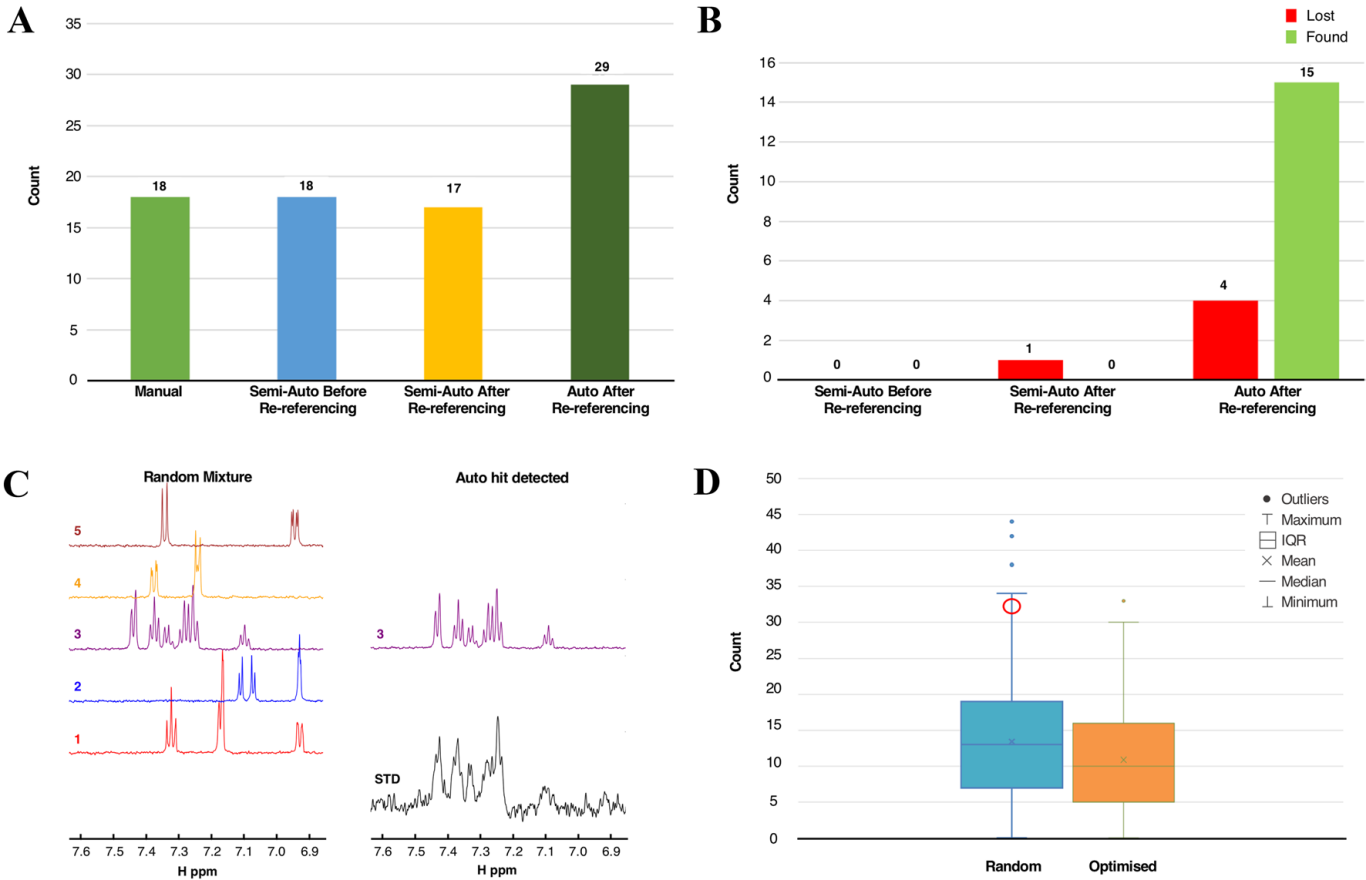
Automated *versus* manual hit detection results. **a** Total number of SpectrumHits obtained by a visual inspection using manually picked peaks (light green bar); SpectrumHits obtained by the hit detection pipeline before and after re-referencing, using the same previously manually picked peaks (blue and yellow bars) and SpectrumHits obtained after re-referencing and automatic peak detection using default parameters (dark green). **b** Newly detected and lost SpectrumHits counts between the four methods. Notably, the automatic approach showed 15 new potential SpectrumHits, which were missed during the manual analysis. **c** Example of STD SpectrumHit and best matched reference (compound 3) for the mixture. Although, all the references in the mixture appeared to have at least one matching peak to the SpectrumHit, the Hit Analysis module was accordingly able to score the references and identify the compound 3 as the top hit. **d** Total number of overlaps for the original randomly created mixtures and for the new optimised mixtures generated by the mixture generation module. Overlaps and other mixture scores were calculated as in NmrMix (Stark et al. 2016). In the red circle the SpectrumHit shown in Fig. 7c is highlighted; it appeared in proximity to the maximum (top horizontal bar) and outliers (coloured dots) as it scored a large degree of overlapping peaks. The rectangular boxes represent the interquartile range (IQR); the “X” symbol inside the IQR represents the mean; long horizontal bar in the middle of the dataset represents the median (second quartile, Q2), the area below and above indicates the first (Q1) and the third quartile (Q3). Q1, Q2 and Q3 are also referred as 25th, 50th, 75th percentile. The maximum is calculated as Q3 + 1.5*IQR and minimum as Q1-1.5*IQR (Galarnyk 2018)

We also tested the performance of our automated STD analysis on the dataset containing 310 experimental STD spectra, acquired for samples in the presence of a biological target and mixture compositions of up to five components. Firstly, for comparison purposes, spectral peaks were manually picked for all available spectra. Using AnalysisScreen’s intuitive tools for visual spectrum inspection (Fig. S8), each of the 310 STD spectra was inspected by comparing it to all 1536 spectra of the reference library. A total of 18 STD spectra displaying STD effects were considered being True Positive SpectrumHits (Fig. 7a). Running the automated matching routine of AnalysisScreen, the same number of SpectrumHits was found (Fig. 7a). However, from the report of the Hit Analysis module we noticed that most of STD spectra were uniformly misaligned to their corresponding reference spectra (Fig. 6a, b) suggesting a potential referencing issue. Referencing problems are commonly present in NMR due to variations in experimental conditions when acquiring screening samples and their reference compound independently (e.g. different spectrometers, temperatures, solvent compositions, etc.). The pipeline, therefore, includes re-reference and global alignment pipes that are capable of automatically detecting and applying shifts to each individual spectrum or, alternatively, setting a specific parameter simultaneously for all spectra. For the dataset under examination, a total shift of 0.0075 ppm was determined (Fig. 6b) and applied to the STDs spectra. Finally, STD spectra were re-matched to the reference data and the hits were re-evaluated.

Ultimately, a complete pipeline, consisting of automatic peak picking, re-referencing, and hit detection pipes was applied to the dataset. A total of 29 SpectrumHits were identified (Fig. 7a). Using the Hit Analysis module, the SpectrumHits were easily inspected and confirmed as True Positive observations whenever they displayed a recognisable signal above the noise. Some of these, however, had very low scores (Fig. S9C–D) and they were missed in the manual visualisation due to simple human oversight. However, four compounds previously flagged during the manual analysis as SpectrumHits were now not found (Figs. 7b and S9A, B), typically because the manual results did not comply with some of our pre-set threshold values, e.g. the spectral Signal-to-Noise Ratio or the peaks were outside the chemical shift matching criteria. Some spectra, in fact, appeared to be very noisy and difficult to interpret even manually. In line with the simulated observations, experimental STD SpectrumHits for peaks with a S/N lower than 1.5 were barely recognisable from the overall noise and, were therefore excluded as True Positive hits. As such, we reinforce the importance of optimising acquisition parameters on a subset of samples to ensure an optimal S/N before the full STD screening study is started.

Inspection of the automated results also showed that some SpectrumHits had multiple matching reference spectra at crucial chemical shift positions, such as the mixture displayed in Fig. 7c. By displaying the total scores for optimised and random mixtures, we identified this element as one of the worst scored, in proximity to the maximum and outliers (Fig. 7d). However, in the optimised mixture, as previously discussed, the corresponding compounds were part of mixtures with significantly less overlap. Therefore, we strongly believe that using the mixture optimisation strategy before-hand would have further facilitated the final hit analysis detection.

## Conclusions

With numerous techniques developed over the years, NMR has been invaluable in all stages of FBDD leading to promising drug-like molecules (Erlanson et al. 2016b). The versatility of NMR spectroscopy has enabled it to tackle all aspects of drug discovery. Starting from the primary screening, NMR ‘chemical resolution’ excels in identifying fragments which bind to the target with very low affinity, including their binding properties (Meyer et al. 2004); NMR also assists in detecting target structural changes upon binding events, elucidating potential known and unknown “hot spots” (Williamson 2013). Lastly, it can be used for determining poses of multiple simultaneously binding fragments, extracting valuable information for the generation of stronger ligands (Sánchez-Pedregal et al. 2005).

Although current techniques provide a multitude of roles and advantages, in everyday practice NMR data analysis can be daunting and time-consuming, generally due to lack of proper tools and uniform data handling practices. Currently, in contrast to AnalysisScreen the commercial Bruker TopSpin (TopSpin) and MestreLab MNova Screen (Peng et al. 2016) software packages unfortunately provide little customisation of individual workflows. Furthermore, hit scoring reports in TopSpin are limited to binary definitions, such as”binding” or “not binding” hits, whereas MNova Screen offers an overall intensity percentage change (Peng et al. 2016). No stand-alone NmrGlue (Helmus and Jaroniec 2013) based scripts for NMR screening data analysis currently exist; however, the routines of this package are also included in the CcpNmr Python environment of AnalysisScreen and thus are directly accessible within the programme, e.g. for incorporation into pipes.

The vast amount of data generated for each screening trial and the lack of freely available software capable of dealing with this data leaves scientists setting up and repeating tiresome operations that could inadvertently lead to human errors. Moreover, users might rely only on qualitative assessments, which can further increase the probability of misinterpreting the data. Here, we introduced CcpNmr AnalysisScreen, a software developed specifically for analysing Fragment-Based Drug Discovery data derived by NMR spectroscopy.

AnalysisScreen is easily able to cope with very large datasets, with a magnitude of tens of thousands of one-dimensional spectral entries and associated metadata, including projects with over 1 million peaks, providing fast and reproducible results. AnalysisScreen is designed in such a way that new user-specific tasks (pipes; Fig. 4) can be easily included in the main program, making it a very flexible platform for custom implementations and bespoke workflows.

We have shown how automated computational tools included in the package, can drastically reduce both the time and bias in analysing the output of NMR screening data compared with manual analysis, including the reduction of False Positive and False Negative observations (Fig. 7). In practice, the manual analysis of a dataset such as the one presented in this manuscript, could take up to several days to complete. In contrast, the whole process can be reduced to minutes for setting and running automated routines, including a final visual assessment of results. We showed how manual analysis can be drastically compromised by alignment issues among experiments. Global automated and manual re-referencing tools are an integral part of the processing pipes of the programme. However, the automated re-alignment of individual peaks within 1D spectra remains a challenging aspect to tackle.

Furthermore, by using the decomposition module as a quick quality control method, the entire reference spectral libraries can be evaluated in seconds before performing the screening analysis (Fig. 3). The principal component analysis has shown its potential also as a CSM screening tool (Namanja et al. 2019), and could be easily employed for assessing 1D relaxations series. Although this strategy can give quicker results, we believe it can reduce the overall sensitivity and hits should also be confirmed by other analysis routines.

AnalysisScreen aims to be the ultimate free non-profit NMR software package able to cover all aspects of fragment-based drug discovery data analysis. As such, it is currently being continuously developed and upcoming releases will include a series of additional processing pipes, such as baseline correction, and automated 1D peak fitting, additional support for automatic analysis of 2D titration series, and new routines for supporting intra- and inter-NOE analysis data analysis used in binding pose elucidation.

We plan for a further enhancement of the mixture generation algorithm by inclusion of additional scoring parameters based on chemical properties of the compounds, such as pK_a_, aggregation probabilities and chemical structural diversities. Furthermore, we aim for an even more exhaustive Hit Analysis module that integrates cheminformatic tools for classifying hits by functional groups and supports the Pan-Assay Interference Compounds (PAINS) filters (Baell and Nissink 2018).

Through the continuing development of CcpNmr AnalysisScreen and its ability to allow for an easy implementation of user-defined functionalities, we believe the platform to be a versatile resource in the data analysis of FBDD data. We ultimately aim for the absence or limited use of user-defined parameters in pipelines to guarantee reliable, reproducible and bias-free outcomes in the primary screen analysis of small-molecule binders by NMR.

## Electronic supplementary material

Below is the link to the electronic supplementary material.

Supplementary file1 (PDF 2400 kb)

## Data Availability

AnalysisScreen release is included in the CcpNmr Analysis 3.0.1 programme suite and is available for downloading for Mac OS, Linux environments, Windows and Virtual Machine from www.ccpn.ac.uk/v3-software/downloads. Documentations, tutorials and user community forums are available at www.ccpn.ac.uk/forums/. The programme is free to use for all non-commercial usage under the LGPL licence.

## Abbreviations

CcpNmr: Collaborative computing project for NMR (software)
FBDD: Fragments based drug discovery
HSQC: Heteronuclear single quantum coherence spectroscopy
K_D_: Dissociation constant
NMR: Nuclear magnetic resonance
PCA: Principal component analysis
NOE: Nuclear overhauser effect
STD: Saturation transfer difference
WaterLOGSY: Water-ligand observation with gradient spectroscopy
TINS: Target immobilised NMR screening
CSP: Chemical shift perturbation
GUI: Graphical user interface
ppm: Part per million
RF: Radio frequency
ROC: Receiver operating characteristic
SMILES: Simplified molecular-input line-entry system
JSON: JavaScript object notation
FDA: Food and drug administration
