# Supplementary material for: CcpNmr AnalysisScreen, a new software programme with dedicated automated analysis tools for fragment-based drug discovery by NMR
Source: J Biomol NMR. 2020 Jul 7;74(10):565–77. doi: 10.1007/s10858-020-00321-1 (PMC7683461; doi:10.1007/s10858-020-00321-1)
Supplement: Supplementary file 1 — Supplementary file1 (PDF 2400 kb) [file 10858_2020_321_MOESM1_ESM.pdf]

CcpNmr AnalysisScreen, a new software programme with dedicated automated analysis tools for fragment-based drug discovery by NMR

Supplementary Information

Luca G. Mureddu, Timothy J. Ragan, Edward J. Brooksbank, Geerten W. Vuister*

Leicester Institute of Structural and Chemical Biology, Department of Molecular and Cell Biology. University of Leicester.

Henry Wellcome Building, Lancaster Road, Leicester LE1 7HN, United Kingdom

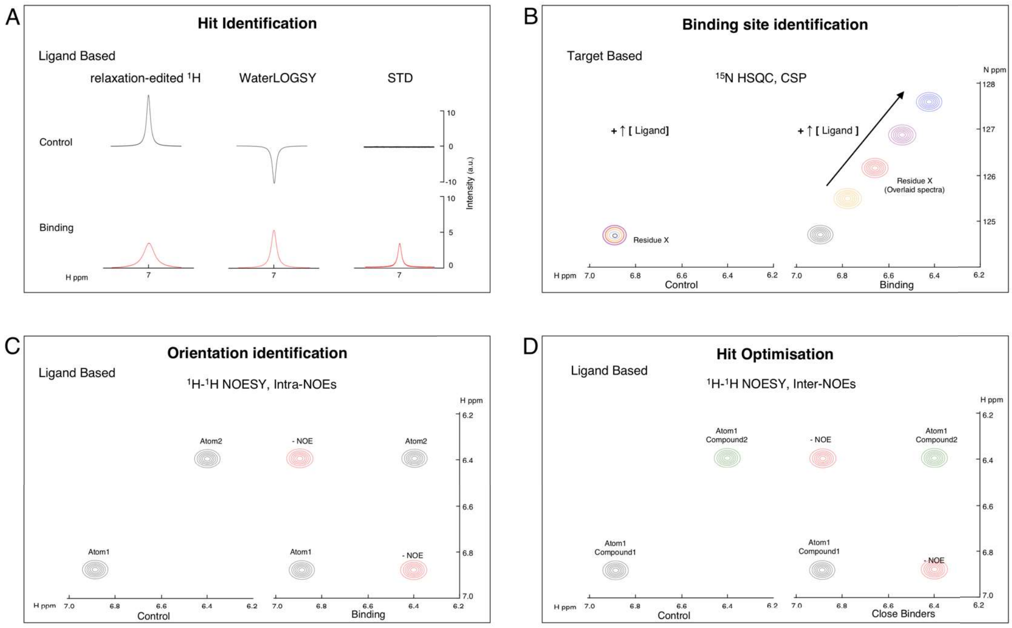

Figure S1. NMR-aided fragment-based drug discovery (FBDD)

Ligand-detected hit identification (**a**), chemical-shift mapping (CSM) binding site identification (**b**), fragment orientation identification and optimisation (**c**-**d**) aided by several ligand-detected NMR techniques[1], mainly Nuclear Overhauser Effects (NOE) experiments, Inter-ligand NOEs for Pharmacophore Mapping (INPHARMA), and Interlined Overhauser effect NMR (ILOE-NMR), but also using specific labelling schemes as in the Selective Labelling STD experiment (SOS-STD)[2]. Note that all chemical shift scales are arbitrary.

a Common ligand-detected hit identification NMR methods and their respective simplified spectral appearances as manifested by recording ligand spectra in absence (control) and presence of the macromolecular target. (Left) The ^{1}H-relaxation-edited control spectrum ligand signals characterised by narrow lines, which broaden and consequently reduce in intensity as result of the increased relaxation rate due to binding the much larger target. (Middle) In the WaterLOGSY experiment saturation of the bulk water is transferred via chemical- and dipolar exchange to the ligand. The binding event is identified by an inversion of the ligand signals compared to the control. (Right) In the saturation-transfer difference (STD) experiment, saturation of target resonances is transferred via dipolar exchange to the ligand. In the STD spectrum, obtain after subtraction of the "off-resonance" from the "on-resonance" spectrum, only the signals of the bound ligand are observed. **b** Chemical shift mapping (CSM) for detection of the ligand binding pockets on the target. In the ^{15}N-HSQC spectrum of a target protein, peaks are commonly used as a distinctive identifying characteristic of individual residues. Upon titration of a small molecule at increasing

concentrations, a ligand binding event will result in a modification of the spectral peak patterns, which can be easily tracked in case of so-called fast exchange. By assignment of the affected peaks to their corresponding residues, and optionally mapping these changes to the protein's three-dimensional structure, it is possible to perform an evaluation of most affected residues in the potential ligand binding pocket (right panel, *Binding*). **c** Schematic overview of a transferred-NOE ^{1}H-^{1}H NOESY spectrum for detection of a ligand pose. In case of binding, the ligand takes on the NOE properties of the target (rotational correlation time, τ_c), showing strong negative NOE, i.e. positive peaks (red) revealing its ligand-bound conformation. In principle, protein-ligand inter-molecular NOEs could be observed as well; however, as the protein concentration is usually much lower compared to the ligand, these NOE will be (much) weaker and difficult to interpret (not shown). **d** Theoretical ^{1}H-^{1}H NOESY spectrum for a sample in the presence of two ligands. Inter-molecular NOEs peaks between molecules are enhanced if they bind in close proximity on the target (red peaks)

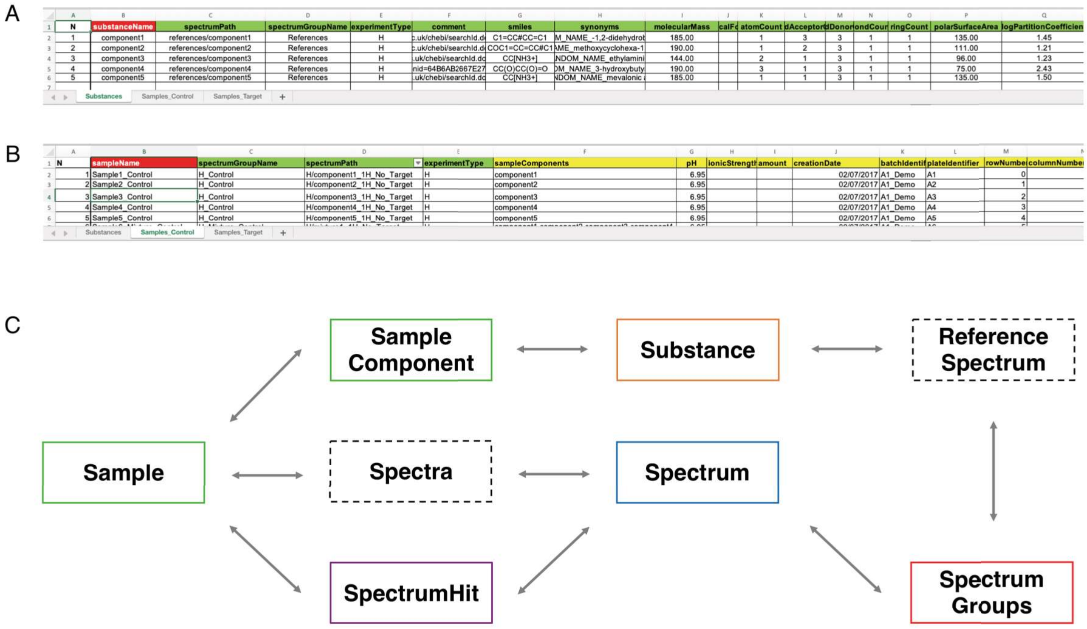

Figure S2. Excel file loading examples

a Substances Excel sheet; Substance, can contain metadata associated with small molecules, including the relative spectrum path for the processed spectrum used as references for a screening trial. The highlighted red column header indicates the mandatory unique substanceName field. **b** Sample Excel sheet; these sheets contain all metadata associated with particular samples and their components. For example, in a screening study the sample might contain multiple spectra recorded under different experimental conditions or using different NMR experiment. **c** Schematic representation of the CcpNmr core objects used in AnalysisScreen. Core objects ensure data is accurately maintained across multiple invocations of the programme; furthermore, the linkage among various items creates a robust data-access strategy for implementation of the data analysis routines available in AnalysisScreen

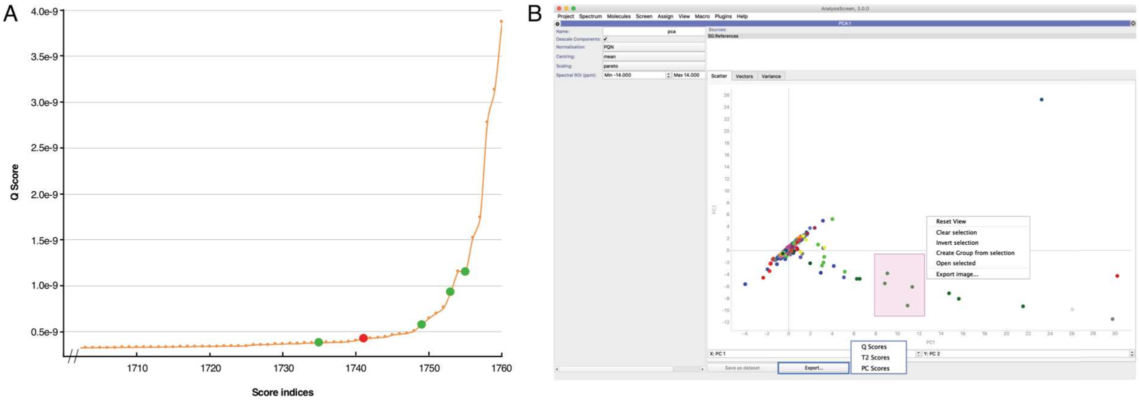

Figure S3. PCA Q-scores and decomposition module

a PCA Q-scores calculated for the experimental library reference dataset of 1760 spectra, displayed in a descending mode for the final 60 values. The highest scoring entries were associated with significant spectral issues, including poor solvent suppression and phasing problems (shown in detail in the main paper Fig. 3, panels c and d). **b** Screenshot of the AnalysisScreen PCA decomposition module. Module-specific settings are displayed on the left; input data section (top area) accepts drag-and-drop of spectra or SpectrumGroup to start the calculation; the central area displays PCA scores, vectors and variance plots

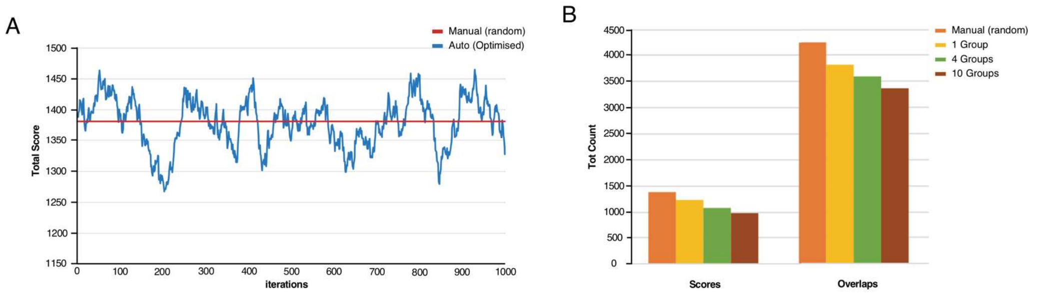

Figure S4. Mixture generation

a Evolution of the total overlap score over 1000 simulated-annealing iterations of the mixtures generation algorithm using 1548 library reference spectra as input data. The red line represents the total overlap score derived from manual randomly created mixtures. **b** Total overlap scores and overlap counts for manual randomly created mixtures (orange), automatically generated and optimised using the whole dataset as input (yellow) or automatically generated on the basis of 4 (green) or 10 groups (brown). See main text for details

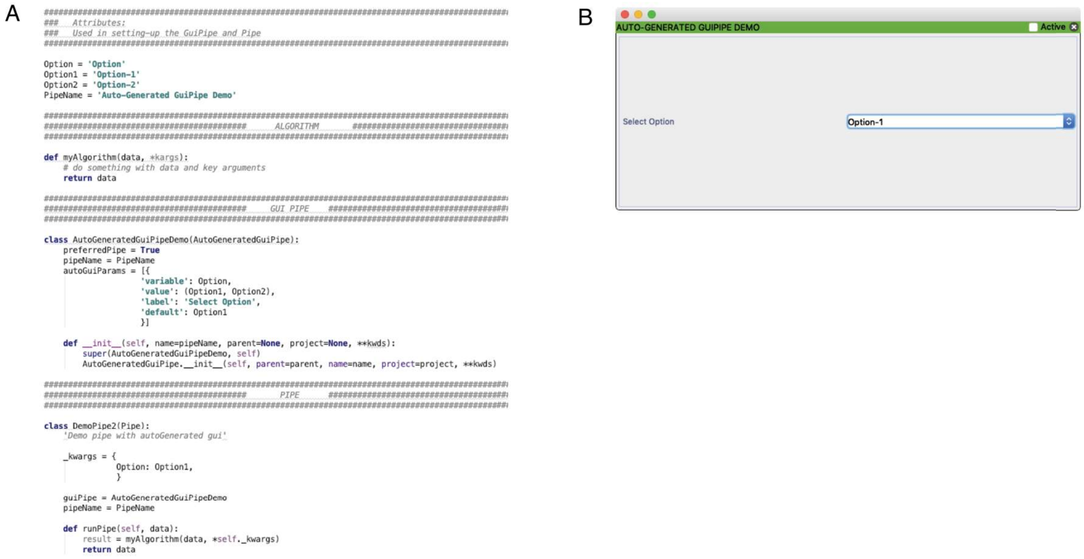

Figure S5. Code template for pipes

a Python source code template for an auto-generated GUI pipe. A Pipe file consists of three sections, the algorithm(s), the GUI class, and the main Pipe class. Graphical User Interface is optional for pipes and pipeline queues can be also run from the Python terminal within the program. Pipes files containing the appropriate code elements become automatically usable in the pipeline module (see also Fig. S6C). **b** A simple demo pipe with an auto-generated GUI generated by the code shown in **a**

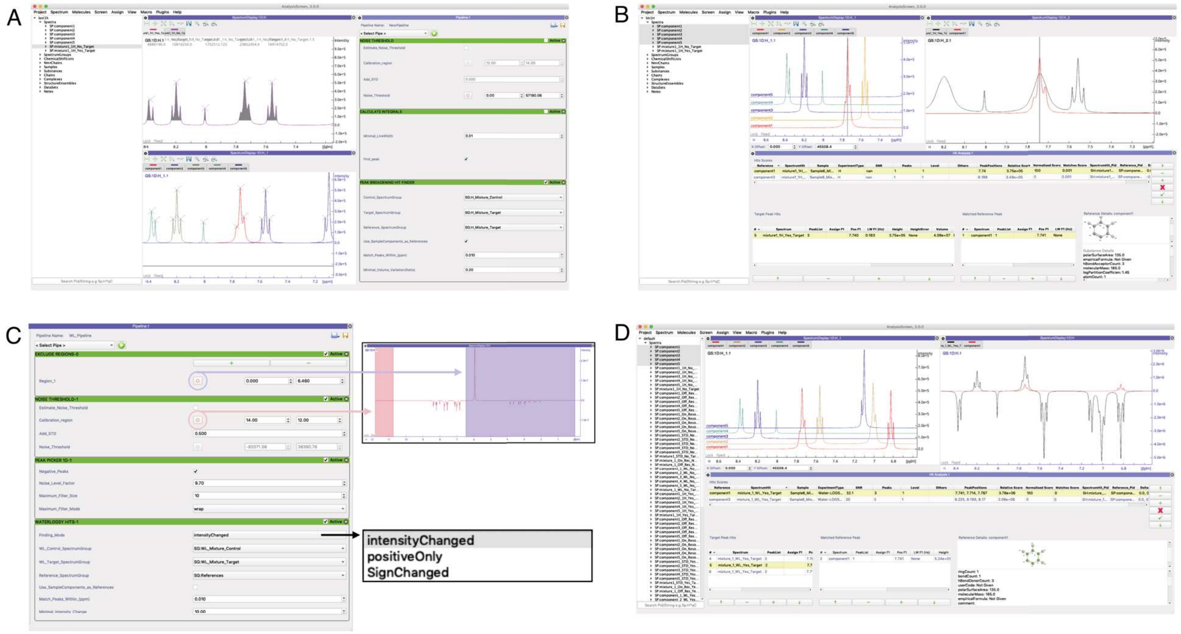

Figure S6. Validation of data analysis pipelines using simulated data

a Simulated ^{1}H relaxation-edited spectra in presence and absence of a target and display of automatically detected integrals (top spectrum display module), library reference spectra (lower spectrum display module), and peak-broadening detection pipeline used in the data analysis (right-hand side). **b** Hit analysis after running a ^{1}H broadening detection pipeline. Top left spectrum display module shows a stack of reference spectra for the sample under examination. Top right spectrum display module shows the ^{1}H spectra for the target experiment and its matched library reference, as calculated by integral value change. The hit analysis module (bottom) displays a summary of various scores for SpectrumHits and matched references properties in the analysis module. **c** WaterLOGSY data analysis pipeline for detecting hits by comparing signal intensity changes and peak matching by chemical shifts. Interactive manipulation of the exclude regions or noise threshold is facilitated by dedicated buttons highlighted in purple and red in the corresponding pipes, respectively. Different calculation modes for the WaterLOGSY hit detection pipe are displayed in the inset box. **d** Hit analysis after running a WaterLOGSY hit detection pipeline. Top left spectrum display module shows the reference spectra for the sample under examination. Top right spectrum display shows the WaterLOGSY spectrum for the target experiment and its matched reference hit. The hit analysis module (bottom) displays a summary of various scores for SpectrumHits and matched references properties in the analysis module

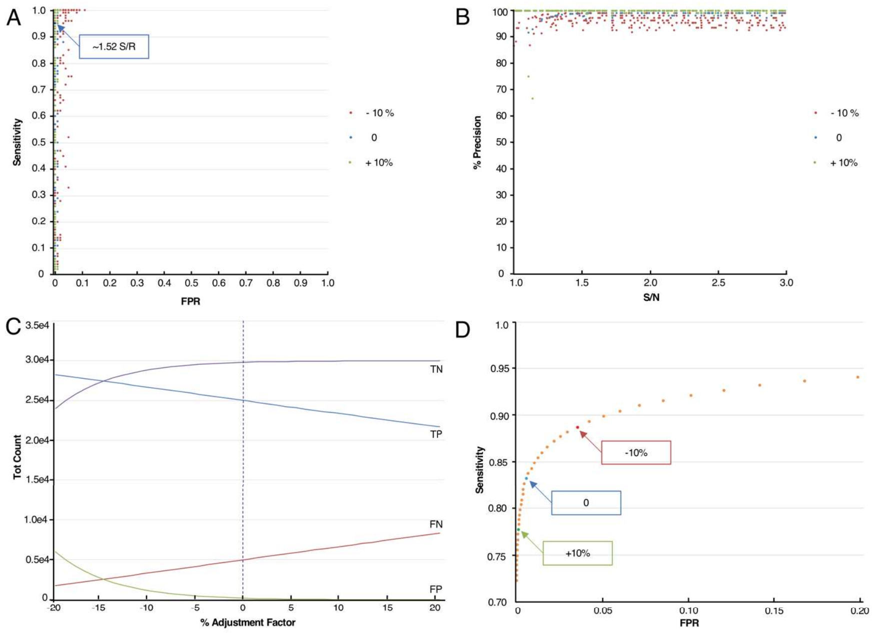

Figure S7. Peak detection statistics

The correctness of the automatically determined peak detection noise threshold value was inspected using receiver operating characteristic (ROC) scoring. It allows to determine the capacity of a binary classifier followed by an adjustment in the threshold value[3]. In the ROC curve the Sensitivity is plotted against the False Positive Rate (FPR). Sensitivity, False Positive Rate, Specificity and Precision, are calculated from the true positive (TP), true negative (TN), false positive (FP), false negative (FN) values. **a** ROC plot for performance of the algorithm at different S/N ratios. Blue arrow indicates the score for a spectrum at ~1.5 S/N. The Sensitivity is calculated as TP/(TP+FN); the False Positive Rate, FPR is calculated as 1-Specificity, where the Specificity is calculated as TN/(TN+FP); **b** Precision of the peak picker for spectra at different S/N ratios. Precision was calculated as TP/(TP+FP). Notably, the precision is badly compromised when lowering the threshold value by 10% (blue data points; -10%) at all S/N ratios. **c** Total count for true positives, true negatives, false positives and false negatives for each run of peak picking using different adjustments of the automatically determined noise threshold value. **d** ROC curve for the total sensitivity and false positive rate, as expected the automatically determined threshold value is located in the most optimal location of the ROC curve, suggesting it can be used reliably in the automatic peak picking routine without need for adjustment

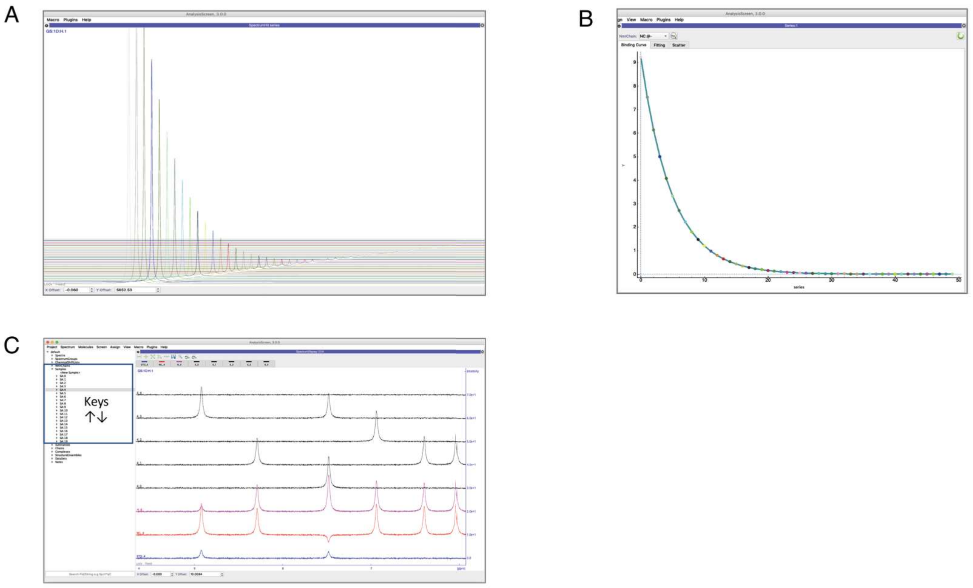

Figure S8 CcpNmr AnalysisScreen tools for spectra manual visual inspection

a Spectrum display module showing a simulated SpectrumGroup comprised of a series of 1D ^{1}H spectra with vertical and horizontal offsets to facilitate a quick inspection. **b** Example of usage of the Chemical Shift Mapping for assessing 1D spectra series. The figure shows a binding curve for the spectra displayed in **a** using peak heights as the input for calculations. **c** The sidebar of AnalysisScreen can be used for easy navigation through data items using up/down keys (indicated by the rectangular box). This option allows to display automatically stacked spectra recorded for the selected sample and their references for a quick and manual visual inspection. NB. this feature is currently enabled using a command line macro

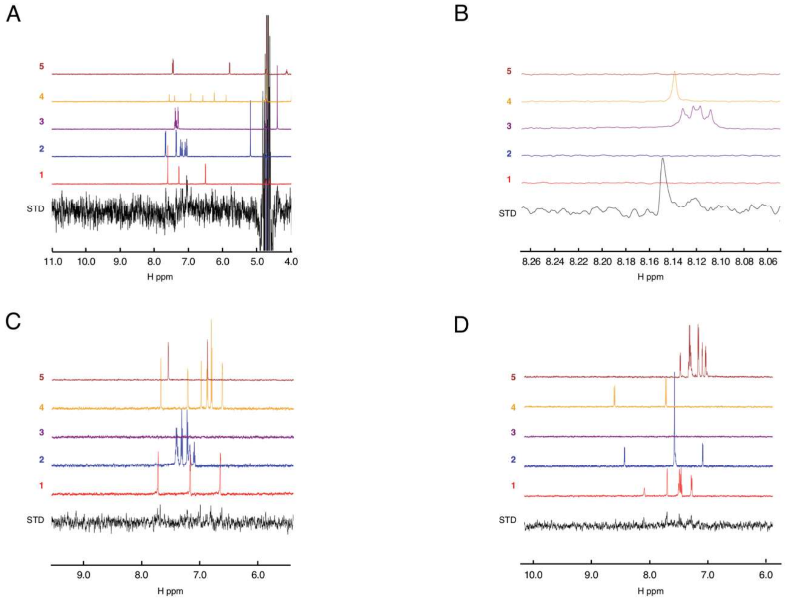

Figure S9 Examples of the analysis of experimental STD spectra

Example experimental STD spectra (black) and five relevant reference spectra are shown. **a** Example of an STD spectrum which was discarded as a true positive on the basis of a low S/N. **b** Example of an STD spectrum with a peak at 8.149 ppm which did not match any of the reference spectra and therefore was excluded as a true positive hit. Examples of STD spectra (**c** and **d**) with very weak matching signals previously not identified during manual inspection

References

1. Sugiki, T., Furuita, K., Fujiwara, T. & Kojima, C. Current NMR techniques for structure-based drug discovery. *Molecules* **23**, 148 (2018).

2. Hajduk, P. J., Mack, J. C., Olejniczak, E. T., Park, C., Dandliker, P. J. & Beutel, B. A. SOS-NMR: A Saturation Transfer NMR-Based Method for Determining the Structures of Protein-Ligand Complexes. *J. Am. Chem. Soc.* **126**, 2390–2398 (2004).

3. Hajian-Tilaki, K. Receiver operating characteristic (ROC) curve analysis for medical diagnostic test evaluation. *Casp. J. Intern. Med.* **4**, 627–635 (2013).
